# Regulation of the type IV pili molecular machine by dynamic localization of two motor proteins

**DOI:** 10.1111/j.1365-2958.2009.06891.x

**Published:** 2009-10-06

**Authors:** Iryna Bulyha, Carmen Schmidt, Peter Lenz, Vladimir Jakovljevic, Andrea Höne, Berenike Maier, Michael Hoppert, Lotte Søgaard-Andersen

**Affiliations:** 1 Department of Ecophysiology, Max Planck Institute for Terrestrial MicrobiologyKarl-von-Frisch Str., 35043 Marburg, Germany; 2 Department of Physics, Philipps-Universität35032 Marburg, Germany; 3 Institute of General Zoology and Genetics, Westfälische Wilhelms Universität48149 Münster, Germany; 4 Institute of Microbiology and Genetics, Georg-August-Universität Göttingen37077 Göttingen, Germany

## Abstract

Type IV pili (T4P) are surface structures that undergo extension/retraction oscillations to generate cell motility. In *Myxococcus xanthus*, T4P are unipolarly localized and undergo pole-to-pole oscillations synchronously with cellular reversals. We investigated the mechanisms underlying these oscillations. We show that several T4P proteins localize symmetrically in clusters at both cell poles between reversals, and these clusters remain stationary during reversals. Conversely, the PilB and PilT motor ATPases that energize extension and retraction, respectively, localize to opposite poles with PilB predominantly at the piliated and PilT predominantly at the non-piliated pole, and these proteins oscillate between the poles during reversals. Therefore, T4P pole-to-pole oscillations involve the disassembly of T4P machinery at one pole and reassembly of this machinery at the opposite pole. Fluorescence recovery after photobleaching experiments showed rapid turnover of YFP–PilT in the polar clusters between reversals. Moreover, PilT displays bursts of accumulation at the piliated pole between reversals. These observations suggest that the spatial separation of PilB and PilT in combination with the noisy PilT accumulation at the piliated pole allow the temporal separation of extension and retraction. This is the first demonstration that the function of a molecular machine depends on disassembly and reassembly of its individual parts.

## Introduction

Type IV pili (T4P) are among the most widespread bacterial cell-surface structures (Pelicic, 2008) and have essential functions in pathogenesis caused by several human pathogens by mediating attachment to and microcolony formation on host cells (Craig *et al.*, 2004), cell motility (Mattick, 2002), biofilm formation (O'Toole and Kolter, 1998; Klausen *et al.*, 2003) and natural transformation (Dubnau, 1999). T4P are highly dynamic and undergo cycles of extension and retraction (Merz *et al.*, 2000; Sun *et al.*, 2000; Skerker and Berg, 2001). The extension/retraction oscillations are central to the diverse functions of T4P. Moreover, in *Myxococcus xanthus* the cell pole at which T4P assemble oscillates in parallel with cellular reversals (Sun *et al.*, 2000; Mignot *et al.*, 2005). Here, we have investigated the mechanisms underlying these two types of T4P oscillations in *M. xanthus.*

Type IV pili are thin (5–8 nm) filaments, several microns in length and typically only composed of the PilA pilin subunit (Craig *et al.*, 2004). T4P systems share a core set of 10 proteins (Pelicic, 2008) and bioinformatic, genetic and biochemical data suggest that T4P proteins form a membrane-spanning protein complex with components in the cytoplasm, inner membrane, periplasm and outer membrane (Ramer *et al.*, 2002; Hwang *et al.*, 2003; Peabody *et al.*, 2003; Crowther *et al.*, 2004; Carbonnelle *et al.*, 2006; Balasingham *et al.*, 2007; Pelicic, 2008). Extension of T4P involves the incorporation of pilin subunits at the base of a pilus (Craig *et al.*, 2006) from a reservoir in the inner membrane (Skerker and Berg, 2001; Morand *et al.*, 2004) and retraction involves the removal and transfer of pilin subunits from the pilus base into the inner membrane (Morand *et al.*, 2004).

Regulation of the T4P extension/retraction oscillations centres on the two motor proteins, PilB and PilT, which are members of the superfamily of secretion ATPases (Craig and Li, 2008). Secretion ATPases are dynamic, hexameric proteins which convert the energy from ATP hydrolysis into translocation of protein and/or DNA over membranes (Savvides, 2007). With the exception of PilT, all T4P proteins are required for T4P extension in otherwise wild-type strains (Craig and Li, 2008). Conversely, PilT is the only protein required for T4P retraction (Merz *et al.*, 2000; Craig and Li, 2008). Genetic and biochemical evidence suggest that PilB and PilT function antagonistically and that the energy for the mechanical work of extending T4P is provided by PilB ATP hydrolysis (Sakai *et al.*, 2001; Chiang *et al.*, 2008; Jakovljevic *et al.*, 2008) and the energy for T4P retraction is provided by ATP hydrolysis by the T4P retraction motor PilT (Satyshur *et al.*, 2007; Chiang *et al.*, 2008; Jakovljevic *et al.*, 2008). How the activities of PilB and PilT are regulated to allow the temporal separation of extension and retraction is not known.

Type IV pili-dependent cell motility, referred to as twitching motility in *Neisseria* species and *Pseudomonas aeruginosa* and as S-motility in *M. xanthus* (Mattick, 2002), occurs when cells are located on a surface and includes three steps: T4P extension, surface adhesion and retraction. While extension does not generate a force sufficient to move a cell, a force exceeding 100 pN per T4P is generated during retractions (Maier *et al.*, 2002; Clausen *et al.*, 2009), and this force is sufficiently large to pull a bacterial cell forward (Merz *et al.*, 2000; Sun *et al.*, 2000; Skerker and Berg, 2001). Individual T4P retract independently of each other (Skerker and Berg, 2001).

*Myxococcus xanthus* cells display two types of morphogenetic cell movements depending on their nutritional status (Leonardy *et al.*, 2008). In the presence of nutrients, colonies form in which cells at the edge spread co-ordinately outward. In the absence of nutrients, the spreading behaviour is constrained and cells aggregate to construct spore-filled fruiting bodies. Both types of morphogenetic cell movements depend on the ability of cells to move and to regulate their motility behaviour. *M. xanthus* cells are rod-shaped and move in the direction of their long axis using two distinct motility systems (Hodgkin and Kaiser, 1979). The S-motility system depends on T4P (Wu and Kaiser, 1995). Although several models have been proposed (Wolgemuth *et al.*, 2002; Mignot *et al.*, 2007; Leonardy *et al.*, 2007), it is currently not known how the second system, referred to as the A-motility system, generates mechanical force. As *M. xanthus* cells move over a surface, they occasionally stop and then resume gliding in the opposite direction, with the old lagging pole becoming the new leading pole (Blackhart and Zusman, 1985). Regulation of the cellular reversal frequency is critical for establishing both types of morphogenetic cell movements (Blackhart and Zusman, 1985). The reversal frequency is regulated by the Frz chemosensory system (Blackhart and Zusman, 1985). In the A-motility system, a cellular reversal involves the Frz-dependent relocation of two polarly localized proteins (Leonardy *et al.*, 2007; Mignot *et al.*, 2007).

*Myxococcus xanthus* contains 5–10 T4P per cell (Kaiser, 1979), which are localized at the leading pole of the cell (Sun *et al.*, 2000; Mignot *et al.*, 2005). A cellular reversal in the T4P-dependent motility system involves a Frz-dependent switch of the pole at which T4P assemble (Sun *et al.*, 2000; Mignot *et al.*, 2005). The mechanism underlying T4P pole-to-pole oscillations has been investigated by localizing three proteins that are required for T4P function: the secretin PilQ forms a gated, multimeric channel for T4P in the outer membrane (Wall and Kaiser, 1999) and was found to localize to both poles using immunofluorescence microscopy (Nudleman *et al.*, 2006). Similarly, Tgl, an outer membrane lipoprotein (Rodriguez-Soto and Kaiser, 1997) required for assembly of PilQ multimers (Nudleman *et al.*, 2006), is present at only one cell pole (Nudleman *et al.*, 2006). Finally, FrzS is located in a cluster at the leading pole and, during a reversal, FrzS relocates to the new leading pole in a Frz-dependent manner (Mignot *et al.*, 2005). A *frzS* mutant still assembles functional T4P (Mignot *et al.*, 2005). Thus, the function of FrzS remains unknown. Importantly, even though T4P and the A-motility system operate independently of each other (Hodgkin and Kaiser, 1979), they generate force in the same direction (Kaiser and Crosby, 1983) and they change polarity synchronously during reversals (Leonardy *et al.*, 2007).

To investigate the mechanisms underlying T4P extension/retraction and T4P pole-to-pole oscillations, we determined the cellular localization of five conserved T4P proteins. These proteins were found to localize in three distinct polar patterns. The localization patterns provide evidence that T4P pole-to-pole oscillations involve the disassembly at the old leading pole and reassembly at the new leading pole of the T4P extension machinery. Moreover, we observed that the molecular motors, PilB and PilT, localize to opposite poles with PilB, the extension motor, predominantly at the leading and PilT, the retraction motor, predominantly at the lagging pole. In addition, PilT displays bursts of accumulation at the leading pole. We suggest that the spatial separation of the two motors, in combination with the occasional accumulation of the retraction motor at the leading pole, constitutes the basis for the temporal separation of extension and retraction.

## Results

We hypothesized that the localization of T4P proteins is a crucial parameter in determining at which pole T4P are assembled in *M. xanthus*. Therefore, we analysed the localization of five T4P core proteins, which in combination localize to three different subcellular compartments.

### PilQ localizes in a bipolar, symmetric pattern

**Fig. 1:**
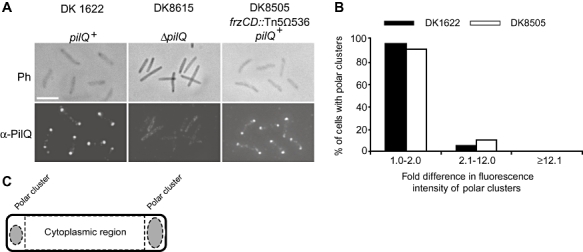
PilQ localizes in a bipolar, symmetric pattern. A. Localization of PilQ by immunofluorescence microscopy. Cells were harvested from exponentially growing cultures, fixed, probed with anti-PilQ antibodies and secondary antibodies, and imaged by fluorescence and phase-contrast microscopy. Top and bottom rows show phase-contrast and fluorescence images respectively. Scale bar: 5 μm. B. Histogram of distribution of PilQ localization patterns. The integrated fluorescence intensities (arbitrary units) of the two background-subtracted polar clusters in a cell were measured and fold differences calculated. Fold differences from 1.0 to 2.0 represent a bipolar, symmetric pattern, differences between 2.1 and 12.0 represent a bipolar, asymmetric pattern, and differences > 12.1 represent a unipolar pattern. C. Schematic of cell indicating the three regions for which fluorescence signals were quantified. Grey ovals indicate polar clusters.

Attempts to construct active mCherry–PilQ fusions were unsuccessful as judged by the inability of the constructs to complement the motility defect of a Δ*pilQ* mutant. Therefore, to localize the outer membrane protein PilQ, we used immunofluorescence microscopy with anti-PilQ antibodies (Fig. S1). As previously observed (Nudleman *et al.*, 2006), PilQ clusters of equal intensities were recognized at both poles in cells fixed directly after growth in suspension (Fig. 1A–C). As T4P-dependent motility only functions in cells on a surface, we determined PilQ localization in cells located on a surface and found that in these cells PilQ was also detected in bipolar, symmetric clusters (data not shown). This localization pattern suggested that the PilQ clusters remain stationary at the poles during reversals. To investigate this hypothesis, PilQ localization was determined in a hypo-reversing *frz* mutant. Also in this mutant, PilQ localized in a bipolar, symmetric pattern both in cells harvested from suspension and in cells harvested from a surface (Fig. 1A and B). We conclude that PilQ localizes in a bipolar, symmetric pattern and that the PilQ clusters likely remain stationary at the poles during reversals.

### PilC localizes in a bipolar, symmetric pattern

**Fig. 2:**
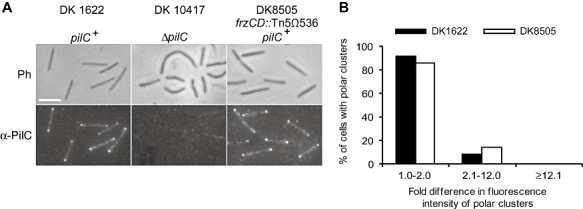
PilC localizes in a bipolar, symmetric pattern. A. Localization of PilC by immunofluorescence microscopy. Cells were harvested from exponentially growing cultures and analysed as described in Fig. 1A using anti-PilC antibodies. Top and bottom rows show phase-contrast and fluorescence images respectively. Scale bar: 5 μm. B. Histogram of distribution of PilC localization patterns. Data are presented as in Fig. 1B.

BfpE, the PilC orthologue of bundle-forming pili in *Escherichia coli*, is an inner membrane protein (Blank and Donnenberg, 2001). Using anti-PilC antibodies (Fig. S2), we confirmed that PilC in *M. xanthus* is also an inner membrane protein (Fig. S3A). As in the case for PilQ, attempts to generate active mCherry/yellow fluorescent protein (YFP)–PilC fusions were unsuccessful. In wild-type cells fixed for immunofluorescence microscopy directly after growth in suspension as well as in cells on a surface, anti-PilC recognized PilC clusters of equal intensities at the two poles (Fig. 2A and B). This pattern was also observed in the hypo-reversing *frz* mutant (Fig. 2A and B). We conclude that PilC is an inner membrane protein that localizes in a bipolar, symmetric pattern and that the PilC clusters likely remain stationary at the poles during reversals.

### PilM localizes in a bipolar, symmetric pattern

Using anti-PilM antibodies (Fig. S3B), we showed that PilM is a soluble protein (Fig. S3A). Because PilM is predicted to contain neither a signal peptide using SignalP (Bendtsen *et al.*, 2004) nor transmembrane helices using TMHMM (Sonnhammer *et al.*, 1998), these data suggest that PilM is a cytoplasmic protein. To localize PilM we expressed a *yfp–pilM* allele from the *pilA* promoter in a Δ*pilM* mutant. YFP–PilM fully corrected the defect in T4P-dependent motility caused by a Δ*pilM* mutation (Fig. S3C). Immunoblots showed that YFP–PilM accumulated at lower levels than PilM protein in wild-type cells and that degradation products corresponding in sizes to those of PilM and YFP accumulated, suggesting that a fraction of YFP–PilM is cleaved near the fusion site (Fig. S3B). Therefore, we localized PilM using YFP–PilM as well as immunofluorescence microscopy.

**Fig. 3:**
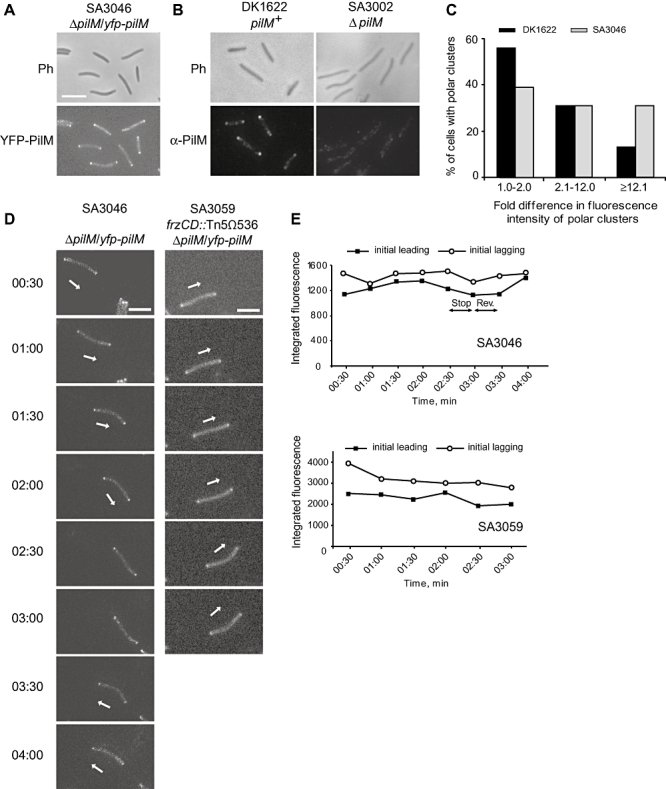
PilM localizes in a bipolar, symmetric pattern. A. Localization of YFP–PilM. Cells were transferred from exponentially growing cultures to a thin 0.7% agar pad on a microscope slide, and imaged by fluorescence and phase-contrast microscopy. Top and bottom rows show phase-contrast and fluorescence images respectively. Scale bar: 5 μm. B. Localization of PilM by immunofluorescence microscopy. Cells were harvested from exponentially growing cultures and analysed as described in Fig. 1A using anti-PilM antibodies. Top and bottom rows show phase-contrast and fluorescence images respectively. C. Histogram of distribution of PilM localization patterns. The data for DK1622 are from immunofluorescence microscopy and for SA3046 from YFP–PilM localization. Data are presented as in Fig. 1B. D. YFP–PilM localization in moving cells. Cells of SA3046 and SA3059 were grown exponentially in CTT, transferred to a thin 0.7% agar pad on a microscope slide, and imaged by fluorescence microscopy at 30 s intervals. Representative cells are shown. The SA3046 cell stopped and reversed from 2:30 to 3:30. Arrows indicate the direction of movement. Scale bar: 5 μm. E. Quantitative analysis of polar YFP–PilM fluorescence signals. Integrated fluorescence intensities (arbitrary units) of the two background-subtracted polar clusters in the cells in (D) plotted as a function of time.

In cells from suspension, YFP–PilM localized in three patterns: bipolar, symmetric, bipolar, asymmetric and unipolar at approximately equal ratios (Fig. 3A and C). In immunofluorescence microscopy of cells from suspension, PilM localized similarly to YFP–PilM (Fig. 3B and C). Because YFP–PilM localizes similarly to that of native PilM, we used YFP–PilM to determine the localization of PilM in moving cells. In moving cells, YFP–PilM localized in a bipolar, symmetric pattern (Fig. 3D and E for a representative cell) and during reversals (*n* = 20) the fluorescence intensity of the polar clusters remained unchanged (Fig. 3D and E for a representative cell). In moving cells of a hypo-reversing *frz* mutant, YFP–PilM localized as in wild type (Fig. 3D and E for a representative cell). We conclude that, upon surface contact, PilM redistributes to a bipolar, symmetric pattern and that the PilM clusters remain stationary at the poles during reversals.

### PilB localizes in three polar patterns

**Fig. 4:**
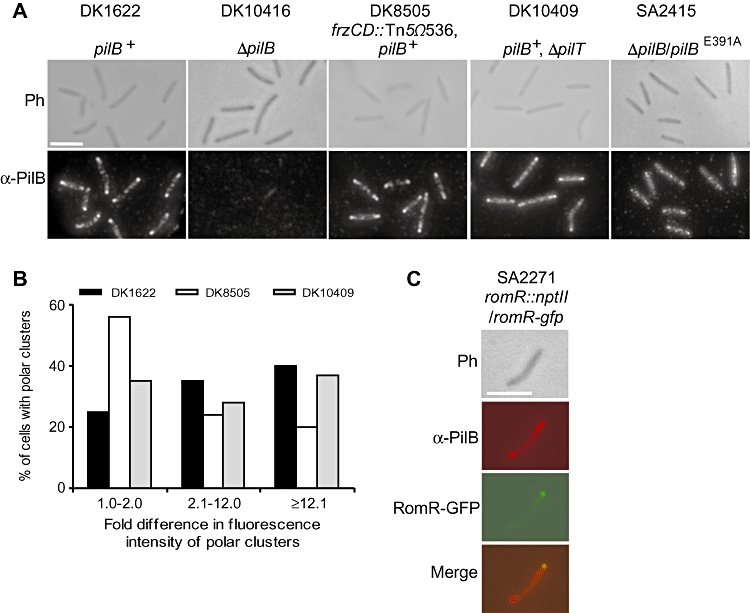
PilB localizes in three polar patterns. A. Localization of PilB by immunofluorescence microscopy. Cells were harvested from exponentially growing cultures and samples were analysed as in Fig. 1A using anti-PilB antibodies. Top and bottom rows show phase-contrast and fluorescence images respectively. Scale bar: 5 μm. B. Histogram of distribution of PilB localization patterns. Data are presented as in Fig. 1B. C. The large PilB cluster localizes opposite to RomR–GFP. Cells were grown, fixed and visualized as in (A).

As reported for *P. aeruginosa* (Chiang *et al.*, 2005), attempts to construct active mCherry/YFP fusion proteins to the cytoplasmic PilB ATPase also failed in *M. xanthus*. In immunofluorescence microscopy using anti-PilB antibodies (Jakovljevic *et al.*, 2008), three distinct PilB localization patterns were observed in cells harvested from suspension and from a surface (Fig. 4A and B): unipolar (40%), bipolar, asymmetric (35%) and bipolar, symmetric (25%). To determine at which pole both the unipolar clusters or the large cluster in cells with bipolar, asymmetric clusters localized, we determined the localization of PilB in a strain containing a RomR–GFP fusion, which localizes in a bipolar, asymmetric pattern with the large cluster at the lagging pole (Leonardy *et al.*, 2007). In these cells, the large PilB cluster was localized at the pole opposite to that containing the large RomR–GFP cluster (Fig. 4C). Thus, as expected, the large PilB cluster is at the leading pole.

The PilB localization patterns are consistent with a model in which PilB is dynamically localized and after a reversal localizes unipolarly at the leading pole. During a reversal period, PilB begins also to build up at the lagging pole giving rise to bipolar, asymmetric and, later, bipolar, symmetric patterns. Finally, during a reversal, PilB at the old leading pole relocates to the new leading pole. This model predicts that in a hypo-reversing *frz* mutant, PilB localization should shift towards a bipolar, symmetric pattern. As predicted, in a hypo-reversing *frz* mutant, a change towards bipolar, symmetric PilB localization was observed in cells in suspension as well as in cells on a surface (Fig. 4A and B).

To determine whether ATPase activity is important for PilB localization, we determined the localization of the PilB^E391A^ mutant protein, which carries a substitution of the highly conserved Glu-391 residue in the Walker B box to Ala. Based on the structures of several secretion ATPases, Glu-391 is important for ATP hydrolysis, whereas ATP binding is predicted to be unaffected by this substitution (Savvides, 2007). Consistently, PilB^E391A^ does not complement the motility defect in a Δ*pilB* mutant and has a strong defect in ATP hydrolysis *in vitro* (Jakovljevic *et al.*, 2008). In strain SA2415 (Δ*pilB*/*pilB*^E391A^), PilB^E391A^ accumulates at levels similar to that of PilB in wild type (Jakovljevic *et al.*, 2008) and is localized in clusters distributed over the entire cell body, including occasionally at the cell poles (Fig. 4A). From these data, we conclude that polar PilB localization depends on ATPase activity.

### PilT relocates between poles during reversals

**Fig. 5:**
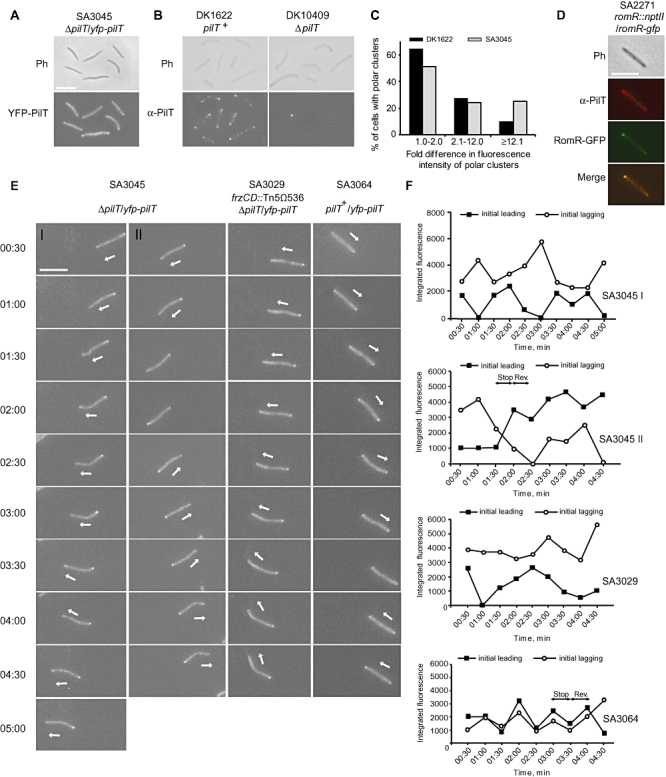
PilT localization is dynamic. A. Localization of YFP–PilT. Cells were transferred from exponentially growing cultures to a thin 1.0% agar pad on a microscope slide, and imaged by fluorescence and phase-contrast microscopy. Top and bottom rows show phase-contrast and fluorescence images respectively. Scale bar: 5 μm. B. Localization of PilT by immunofluorescence microscopy. Cells were harvested from exponentially growing suspension cultures and samples analysed as in Fig. 1A using anti-PilT antibodies. Top and bottom rows show phase-contrast and fluorescence images respectively. C. Histogram of distribution of PilT localization patterns. The data for DK1622 are from immunofluorescence microscopy and for SA3045 from YFP–PilT localization. Data are presented as in Fig. 1B. D. The large PilT cluster colocalizes with the large RomR–GFP cluster. Cells were grown, fixed and visualized as in (B). E. YFP–PilT localization in moving cells. Cells of SA3045, SA3029 and SA3064 were grown exponentially in CTT, transferred to a thin 0.7% agar pad on a microscope slide, and imaged by fluorescence microscopy at 30 s intervals. Representative cells are shown. The SA3045 cell in panel II reversed from 1:30 to 2:30 and the SA3064 cell reversed from 3:30 to 4:00. White arrows indicate the direction of movement. Scale bar: 3 μm. F. Quantitative analysis of polar YFP–PilT fluorescence signals. Integrated fluorescence intensities (arbitrary units) of the two background-subtracted polar clusters in the cells in (E) were plotted as a function of time.

To localize PilT, a *yfp–pilT* allele was expressed from the *pilA* promoter in a Δ*pilT* mutant. YFP–PilT fully corrected the defect in T4P-dependent motility caused by the Δ*pilT* mutation (Fig. S4A). Immunoblot analysis showed that YFP–PilT accumulated at a level similar to that of PilT protein in wild-type cells (Fig. S4B); however, a degradation product was also detected (Fig. S4B, grey arrow) suggesting that a fraction of YFP–PilT is cleaved. Therefore, we localized PilT using YFP–PilT as well as by immunofluorescence microscopy. In cells analysed directly from suspension YFP–PilT (Fig. 5A and C) as well as PilT by immunofluorescence microscopy (Fig. 5B and C) predominantly localized in bipolar, symmetric clusters. Unexpectedly, in cells with a unipolar cluster or with bipolar, asymmetric clusters, the large PilT cluster was at the lagging cell pole as observed using RomR–GFP as a marker for the lagging pole (Fig. 5D). Because YFP–PilT and native PilT localize similarly, we determined the localization of PilT in moving cells using YFP–PilT.

In moving cells, YFP–PilT localized in a unipolar or in a bipolar, asymmetric pattern (Fig. 5E and F for representative cells). Importantly, the large YFP–PilT cluster in these cells was also localized at the lagging pole. The YFP–PilT signal at the leading cell pole varied greatly over time in individual cells and occasionally disappeared completely. Thus, PilT shifts localization from a predominantly bipolar, symmetric pattern in cells in suspension to an asymmetric polar pattern in moving cells.

To resolve whether PilT localization changes during reversals, YFP–PilT localization was followed in 20 reversing cells. All reversals were accompanied by relocation of the large YFP–PilT cluster from the old lagging pole to the new lagging pole (Fig. 5E and F panel II for a representative cell; Fig. S4C and D for a cell displaying several reversals). Dynamic localization of YFP–PilT was also observed during reversals in the presence of 25 μg ml^−1^ chloramphenicol (data not shown). Thus, YFP–PilT relocation involves transfer of YFP–PilT between the cell poles. In a hypo-reversing *frz* mutant (Fig. 5E and F for a representative cell), YFP–PilT also localized with the large cluster at the lagging pole and with bursts of accumulation at the leading cell pole (Fig. 5E and F). These cells did not reverse, and the large YFP–PilT cluster did not relocate between the poles. Thus, Frz is required for relocating the PilT clusters during reversals.

**Fig. 6:**
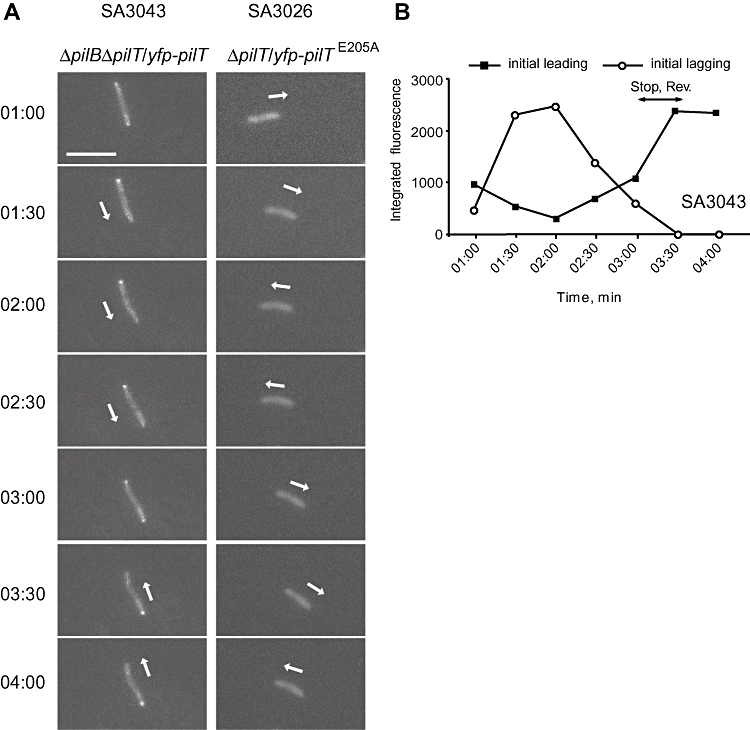
PilT localization is independent of PilB and dependent on ATPase activity. A. Cells of SA3043 and SA3026 were grown exponentially in CTT, transferred to a thin 0.7% agar pad on a microscope slide, and imaged by fluorescence microscopy at 30 s intervals. Representative cells are shown. The SA3043 cell reversed from 3:00 to 3:30. White arrows indicate the direction of movement. Scale bar: 3 μm. B. Quantitative analysis of polar YFP–PilT fluorescence signals. Integrated fluorescence intensities (arbitrary units) of the two background-subtracted polar clusters in the SA3043 cell in (A) plotted as a function of time.

To determine whether ATPase activity is important for PilT localization, the localization of a YFP–PilT protein that carries the substitution Glu-205 to Ala in the Walker B box was examined. As in the case of Glu-391 in PilB, Glu-205 is important for ATP hydrolysis whereas ATP binding is predicted to be unaffected. Consistently, PilT^E205A^ does not complement the motility defect in a Δ*pilT* mutant and has a strong defect in ATP hydrolysis *in vitro* (Jakovljevic *et al.*, 2008). In SA3026 (Δ*pilT/yfp–pilT*^E205A^), YFP–PilT^E205A^ accumulated at levels similar to that of YFP–PilT in SA3045 (Δ*pilT/yfp–pilT*) (Fig. S4B). YFP–PilT^E205A^ localized diffusely over the entire cell body (Fig. 6A). Thus, polar localization of PilT depends on ATPase activity.

The observations that PilB predominantly localizes to the leading cell pole and PilT predominantly to the lagging cell pole suggested that correct PilB and PilT localization could involve a mutually exclusive mechanism in which PilB would at least partially inhibit accumulation of PilT at the leading cell pole and PilT would at least partially inhibit PilB accumulation at the lagging cell pole. To test this hypothesis, we determined the localization of PilB in DK10409 (Δ*pilT*) using immunofluorescence and YFP–PilT in SA3043 (Δ*pilB,*Δ*pilT/yfp–pilT*). In DK10409 PilB localized in a pattern similar to that in wild type (Fig. 4A and B). Likewise, YFP–PilT in moving cells of SA3043 localized in a pattern similar to that of YFP–PilT in moving cells of SA3045 (Δ*pilT/yfp–pilT*) (Fig. 6A and B). Thus, PilB and PilT localize to the cell poles independently of each other.

### PilT turns over in the polar PilT clusters

Given that the PilT ATPase is the motor that powers T4P retraction, it was surprising that the large PilT cluster was at the lagging pole in moving cells. Between reversals the YFP–PilT signal at the leading cell pole varies giving rise to either a unipolar or a bipolar, asymmetric pattern (Fig. 5E and F). We hypothesized that turnover of PilT molecules in the PilT clusters between reversals and on a timescale much shorter than that of an average reversal period (15 min under our conditions) could result in the noisy accumulation of PilT at the leading pole. At the leading pole, PilT would be able to interact with the T4P machinery at the base of a T4P, thus forming a retraction machinery.

**Fig. 7:**
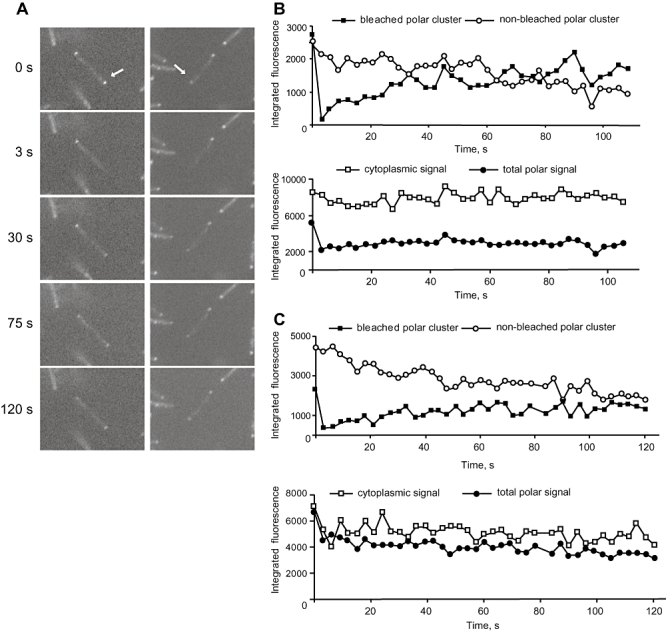
Polar PilT clusters are in a dynamic equilibrium. A. Successive fluorescence images of YFP–PilT cells (SA3045) before (0 s) and after bleaching (3 s to 120 s) of a polar region. Bleaching was for 1 s. Bleached polar regions are indicated by arrows. In the cell on the left, the polar region with the large YFP–PilT cluster was bleached, and in the cell on the right, the polar region with the small YFP–PilT cluster was bleached. B and C. Quantitative analysis of recovery and loss of YFP–PilT fluorescence signals. Integrated fluorescence intensities (arbitrary units) of the polar clusters, the total polar signal (sum of the two polar cluster signals) and the total cytoplasmic signal in the cells in (A) were plotted as a function of time. Data in (B) and (C) are from the cell in the left and right panels in (A) respectively.

To investigate PilT turnover in the polar YFP–PilT clusters we conducted a Fluorescence Recovery After Photobleaching (FRAP) experiment with cells on a surface. In anti-PilT immunofluorescence microscopy of wild-type cells 58 ± 8% of the total fluorescence is detected in polar clusters (Fig. 5B), whereas in Δ*pilT*/*yfp–pilT* cells, only 24 ± 4% of the total fluorescence is detected in polar clusters (Fig. 5A). We attribute the increased cytoplasmic signal in the Δ*pilT*/*yfp–pilT* cells to degradation of YFP–PilT with the formation of a fluorescent degradation product that does not localize polarly (Fig. S4B). Therefore, in the FRAP experiments, the background fluorescence was reduced by pre-bleaching the cytoplasmic region between the polar clusters (Fig. 1C). Subsequently, an area encompassing a polar region was bleached for 1 s and then fluorescence recovery and fluorescence loss followed for 120 s. In these experiments, we specifically analysed cells with two PilT clusters to determine if the two clusters were in a dynamic equilibrium. After bleaching of a polar region, the fluorescence signal at the bleached pole showed recovery and stabilized after 50 ± 10 s. Importantly, the fluorescence signal at the non-bleached pole was lost in a manner resembling the increase in the fluorescence signal at the bleached pole (Fig. 7A–C). The results were similar regardless of whether a large cluster (Fig. 7A and B) or a small cluster (Fig. 7A and C) was bleached. Consistently, the total polar signal and the fluorescence signal from the cytoplasmic region in both types of experiments remained relatively constant (Fig. 7B and C). Similar data were obtained without pre-bleaching of the cytoplasm (data not shown). Thus, there is a correlation between recovery of fluorescence at the bleached pole and loss of fluorescence at the non-bleached pole. From these analyses we conclude that PilT molecules undergo turnover in both PilT clusters and that PilT is in a dynamic equilibrium and is continuously exchanged between the two polar clusters.

**Fig. 8:**
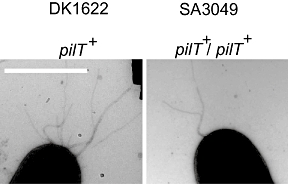
Overexpression of PilT reduces the number of T4P. Cells from exponentially growing cultures of the indicated strains were directly transferred to a grid, stained with 2% (w/v) uranyl acetate and visualized using transmission electron microscopy. Scale bar, 1.0 μm. Wild-type cells contain 6.5 ± 3.0 T4P per piliated pole and *pilT*^+^/*pilT*^+^ cells 3.4 ± 2.0 T4P per piliated pole (*P* = 0.0005).

If T4P retraction depends on the noisy accumulation of PilT at the leading cell pole, we predicted that increased accumulation of PilT at the leading pole should result in cells with fewer T4P. To test this prediction, strains that overproduced PilT were constructed by expressing *pilT*^+^ or *yfp–pilT* from the *pilA* promoter in wild-type cells. Immunoblot analysis showed that PilT accumulated at a twofold higher level in *pilT*^+^/*pilT*^+^ cells compared with wild type and that PilT plus YFP–PilT in the *pilT*^+^/*yfp–pilT* cells accumulated at a twofold higher level than PilT in wild type (Fig. S4B). In moving *pilT*^+^/*yfp–pilT* cells, YFP–PilT localized in a bipolar, symmetric pattern suggesting an increased accumulation of PilT at the leading pole (Fig. 5E and F). Using transmission electron microscopy to visualize T4P, we found that wild type (*n* = 20) as well as *pilT*^+^/*pilT*^+^ cells (*n* = 20) assembled T4P in unipolar patterns (Fig. 8 for representative cells). Importantly, wild-type cells contained significantly more T4P than *pilT*^+^/*pilT*^+^ cells (6.5 ± 3.0 and 3.4 ± 2.0 T4P per piliated pole respectively; *P* = 0.0005). Thus, an increase in the concentration of PilT at the leading pole correlates with a decrease in the number of T4P.

## Discussion

Type IV pili undergo two types of oscillations in *M. xanthus*, extension/retraction oscillations between reversals and pole-to-pole oscillations during reversals. To address the mechanisms underlying these oscillations, we determined the localization of five core T4P proteins. The localization patterns of these five proteins provide evidence that both types of oscillations depend on dynamic disassembly/reassembly of the T4P machinery.

**Fig. 9:**
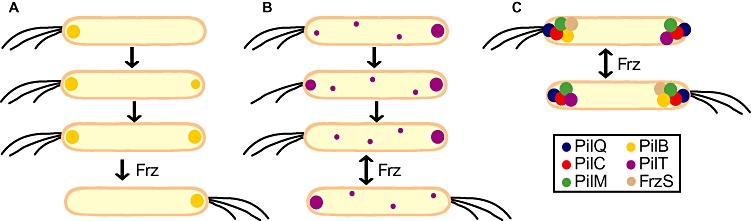
T4P function depends on disassembly and reassembly processes. A. Model for PilB localization. Immediately after a reversal, PilB is unipolarly localized at the T4P pole. During a reversal period, PilB also builds up at the non-piliated pole giving rise to a bipolar, asymmetric and a bipolar, symmetric pattern. In response to Frz activity, PilB localization is reset to a unipolar pattern with PilB at the new leading pole. B. Model for PilT localization. The majority of PilT is localized to a large cluster at the lagging cell pole with some PilT in the cytoplasm. PilT is rapidly turned over in the cluster resulting in the stochastic accumulation of PilT at the leading cell pole followed by retraction of T4P. In the absence of PilT at the leading cell pole, T4P extension is catalysed by PilB. In response to Frz activity, the PilT cluster at the lagging cell pole relocalizes to the new lagging cell pole. C. Pole switching of T4P involves the disassembly and reassembly of the T4P molecular machine. PilQ, PilC and PilM are present in symmetric clusters that remain stationary at the poles during cell reversals. PilB and FrzS are predominantly at the leading pole, PilT predominantly at the lagging pole, and these three proteins relocalize during a reversal in response to Frz activity.

Our data provide evidence that three of these proteins, PilQ in the outer membrane, PilC in the inner membrane and PilM in the cytoplasm, localize in bipolar, symmetric clusters and that these clusters remain stationary at the cell poles during reversals (Fig. 9C).

In contrast, the localization patterns observed for the PilB ATPase in wild-type and hypo-reversing *frz* mutant cells using immunofluorescence microscopy suggest a model in which PilB localization is dynamic between, as well as during, reversals (Fig. 9A). According to this model, PilB is localized unipolarly at the leading pole after a reversal. During a reversal period, PilB begins to accumulate at the lagging pole initially giving rise to a bipolar, asymmetric and later to a bipolar, symmetric pattern. Finally, when a cell reverses, PilB at the old leading pole relocates to the new leading pole, thus, giving rise to a unipolar pattern. Because a reversal period (15 min under our conditions) is much shorter than the generation time of *M. xanthus* (4–5 h under our conditions), only ∼5% of the total PilB protein is synthesized during a reversal period. Moreover, the half-life of PilB is on the order of 200 min (data not shown). These observations suggest that most of PilB that accumulates at the lagging pole would derive from PilB released from the cluster at the leading pole. According to this model, the Frz system, which regulates the reversal frequency, functions to reset PilB localization from a bipolar, symmetric to a unipolar pattern. In a *frz* mutant, this resetting mechanism would be absent, and therefore the bipolar, symmetric localization pattern of PilB is predominantly observed in the hypo-reversing *frz* mutant. Accordingly, the Frz system is not a direct pole-targeting determinant of PilB, but it is required to reset the system to asymmetry.

We directly observed that PilT localization is dynamic between and during reversals (Fig. 9B). The PilT ATPase localizes in two polar patterns in moving cells: unipolar and bipolar, asymmetric. Unexpectedly, the large PilT cluster localizes to the lagging pole. In individual cells, the cluster at the leading pole was highly variable and appeared and disappeared over time (this observation is discussed further below). Notably, in a hypo-reversing *frz* mutant, PilT asymmetry was maintained. Thus, PilT does not progressively accumulate at the leading pole during a reversal period as suggested for PilB at the lagging pole. Importantly, during reversals, the large YFP–PilT cluster relocates from the old lagging to the new lagging cell pole in a Frz-dependent manner.

The localization patterns of PilQ, PilC, PilM, PilB, PilT and FrzS (Mignot *et al.*, 2005) suggest that T4P proteins can be divided into two groups (Fig. 9C). One group is represented by PilQ, PilC and PilM, and these proteins are localized in bipolar, symmetric clusters, which remain stationary at the poles during reversals. The second group is represented by PilB, PilT and FrzS, and these proteins oscillate between the poles during reversals. PilB and FrzS are predominantly found at the leading pole and PilT predominantly at the lagging pole. On the basis of these data, we suggest that pole-to-pole oscillations of T4P involve the disassembly at the leading pole of the T4P extension machinery with the release of PilB and FrzS and reassembly at the new leading pole of the T4P extension machinery with the binding of PilB and FrzS to the pre-assembled stationary T4P proteins. In parallel, PilT is released from the old lagging pole and relocates to the new lagging pole. PilQ, PilC and PilM are localized to the outer membrane, inner membrane and cytoplasm respectively. We speculate that these T4P proteins interact to generate stationary membrane-spanning protein complexes that may include other T4P proteins. The complexes at opposite poles would be activated in an alternating pattern depending on the localization of the PilB and PilT motor proteins. To our knowledge, this is the first time that the function of a molecular machine has been shown to depend on the dynamic disassembly and reassembly of its individual parts.

PilB, PilT and FrzS may not be the only T4P proteins that oscillate between the poles during a reversal. Notably, in cells lacking PilT, PilB localizes as in wild-type cells and some of these cells have PilB clusters at both poles. Nevertheless, these cells only contain T4P at one pole (Wu *et al.*, 1997; Jakovljevic *et al.*, 2008), suggesting that an as yet to be identified protein(s) required for T4P assembly also relocates during reversals. These observations also demonstrate that PilT does not simply function to retract T4P at the lagging pole. The outer membrane lipoprotein Tgl localizes in a unipolar pattern (Nudleman *et al.*, 2006); however, it is not known whether Tgl localization is dynamic. We are currently analysing at which pole Tgl is localized and if Tgl localization is dynamic. Likewise, it remains an open question whether the PilA pilin subunits relocate between the poles during a reversal. The dynamic localization of FrzS, PilB and PilT during reversals is induced by the Frz system; however, it remains to be elucidated how Frz induces relocation of these proteins.

Type IV pili function depends on the temporal separation of extension and retraction. It has remained an open question how the activities of the PilB and PilT ATPases are regulated to allow the temporal separation of extension and retraction. Our PilB and PilT localization data provide a solution to this question. As expected, PilB asymmetry was strongly biased towards the leading pole where it would energize T4P extension. Unexpectedly, the large PilT cluster was localized at the lagging pole with bursts of accumulation at the leading pole. In FRAP experiments we determined that PilT molecules in the polar clusters are rapidly turned over and that the PilT molecules in the polar clusters are in a dynamic equilibrium on a timescale that is much shorter than a reversal period. Based on a detailed quantitative analysis of the FRAP data (see below), we propose that the dynamic exchange of PilT proteins between the poles allows PilT to occasionally accumulate at the leading pole. Once at the leading pole, PilT would interact with a T4P machinery to energize retraction. One scenario for how PilT may promote retractions is that it could displace PilB at the base of a T4P. Alternatively, PilT and PilB could interact in parallel with a T4P machinery. The observation that targeting of PilB and PilT to opposite poles does not involve a mutually exclusive mechanism suggests that PilB and PilT interact in parallel with the T4P machinery.

In the absence of evidence supporting active transport of PilT between the poles, we analysed the diffusion of PilT in the cytoplasm and the binding transitions to the leading and lagging pole. For a freely diffusible cytoplasmic protein with a molecular mass of 72 kDa, the experimentally determined diffusion coefficient *D*_*a*_ is 2.5 ± 0.6 μm s^−1^ (Elowitz *et al.*, 1999). YFP–PilT has a molecular mass 67.6 kDa and, thus, is expected to have a diffusion coefficient on the order of 2.5 μm^2^ s^−1^. From the Stokes-Einstein relation it then follows that the diffusion coefficient for a YFP–PilT hexamer is 1.5 μm^2^ s^−1^. Using these diffusion coefficients, PilT monomers and hexamers would need ∼7 s and ∼12 s, respectively, to relocate between the poles by diffusion. However, a quantitative analysis of the FRAP data show that the mean waiting time for binding of PilT is on the order of 80 s (see *Supporting information*). Therefore, after release from one of the poles PilT protein takes, on average, 80 s until it binds again to one of the poles. Thus, the dynamic exchange of PilT between the two poles is not diffusion-limited but is likely limited by the on- and off-events, i.e. binding and unbinding from the poles. In fact, as shown in *Supporting information*, dissociation from the poles is slower than association. The half-life of the small or large cluster, defined as the time needed for 50% of the bound PilT to be replaced by PilT from the opposite pole, is 40 ± 20 s. Thus, the clusters are highly dynamic structures. From quantitative PilT immunoblots (data not shown), we estimate that individual cells contain 1800 PilT molecules which is equivalent to 300 PilT hexamers. Given that 58 ± 8% of PilT is bound at the poles, approximately 1080 molecules (180 hexamers) are at the poles and 720 molecules (120 hexamers) are in the cytoplasm. It is not known whether PilT in the polar clusters and in the cytoplasm is present as monomers or hexamers. Regardless, given these low numbers of protein and slow binding and unbinding dynamics of PilT at the poles, the accumulation of PilT at the leading pole is expected to be heavily influenced by the stochasticity of the single protein-binding processes. Therefore, PilT accumulation at the leading pole is expected to be noisy and show variation over time.

A prediction from this model is that the overproduction of PilT should result in cells with fewer T4P. Consistently, we observed that a twofold increase in the accumulation of PilT results in cells with approximately twofold fewer T4P. Therefore, we suggest that the spatial separation of PilB and PilT in combination with the noisy accumulation of PilT at the leading pole allows the temporal separation of extension and retraction. According to this model, PilB in the absence of PilT at the leading pole is able to energize T4P extension. The occasional accumulation of PilT at the leading pole would allow PilT to intermittently cause retractions. Consistent with this model, it was observed that T4P are retracted at a reduced frequency in a *Neisseria gonorrhoea* mutant that accumulates reduced levels of PilT (Maier *et al.*, 2004). Interestingly, an *M. xanthus* mutant lacking PilT contains the same number of T4P as wild type (Jakovljevic *et al.*, 2008), suggesting that in wild-type T4P assembly activity is occurring close to the maximum rate and that retraction (PilT activity) is low.

Polar localization appears to be a shared property of T4P secretion ATPases. In bundle-forming pili of enteropathogenic *E. coli*, the PilT orthologue BfpF was shown by immunofluorescence microscopy to localize unipolarly (Hwang *et al.*, 2003). A non-active PilB–YFP and an active YFP–PilT both localize in bipolar symmetric patterns in *P. aeruginosa*, whereas an active YFP–PilU fusion (PilU is a paralogue of PilT and required for T4P retraction in *P. aeruginosa*) localizes in a unipolar pattern at the piliated pole (Chiang *et al.*, 2005). In these analyses, cells were observed under conditions where they would not display T4P-dependent motility. In *M. xanthus*, PilT localizes in a bipolar, symmetric pattern under similar conditions but redistributes into the asymmetric pattern when cells are placed on a surface that allows T4P-dependent motility. Thus, it remains unclear whether the model suggested for *M. xanthus* which allows the temporal separation of PilB and PilT activities is applicable to *P. aeruginosa.*

Secretion ATPases form hexameric structures that undergo large nucleotide-dependent conformational changes upon ATP binding and hydrolysis (Savvides, 2007; Craig and Li, 2008). We found that mutant PilB and PilT proteins that can bind, but not hydrolyse ATP, no longer localize to the cell poles suggesting either that polar targeting of PilB and PilT depends on a particular protein conformation or that polar localization is actively maintained and depends on ATP hydrolysis. Interestingly, in *P. aeruginosa*, mutant PilT and PilU proteins that contain amino acid substitutions similar – but not identical – to those described here for PilB and PilT still display normal polar localization (Chiang *et al.*, 2008), suggesting that the mechanisms by which these proteins are targeted to the poles are different in different species.

In bacteria, pole-to-pole oscillations of proteins are involved in processes as diverse as regulation of cell division (Raskin and de Boer, 1999; Thanbichler and Shapiro, 2006), monitoring of cytokinesis (Matroule *et al.*, 2004) and polarity of motility machines (Mignot *et al.*, 2005; 2007; Leonardy *et al.*, 2007). These processes all relate to the spatial organization of bacterial cells suggesting that pole-to-pole oscillations of proteins is a highly capable method to regulate and monitor processes that concern the spatial organization of bacterial cells.

## Experimental procedures

### Strain and plasmid constructions, growth and motility assays, transmission electron microscopy, cell fractionation, antibody generation and immunoblotting

These methods are described in *Supporting information*. *M. xanthus* strains are listed in Table 1.

**Table 1:** *M. xanthus* strains used in this work.

Strain or plasmid	Relevant characteristics^a^	Source or reference
DK1622	Wild type	Kaiser (1979)
DK8615	Δ*pilQ*	Wall *et al.* (1999)
DK8505	*frzCD*::Tn*5lac*Ω536	Sager and Kaiser (1994)
DK10417	Δ*pilC*	Wu and Kaiser (1997)
DK10416	Δ*pilB*	Wu and Kaiser (1997); Jakovljevic *et al.* (2008)
DK10409	Δ*pilT*	Wu and Kaiser (1997); Jakovljevic *et al.* (2008)
SA2271	*romR::nptII*/P_*nat*_*–romR–gfp* (pSH1208)	Leonardy *et al.* (2007)
SA2415	Δ*pilB*/P_pilA_*–pilB*^E391A^ (pSL105BWalkerB)	Jakovljevic *et al.* (2008)
SA3002	Δ*pilM*	This work
SA3026	Δ*pilT/*P_pilA_*–yfp–pilT*^E205A^ (pIB74)	This work
SA3027	*frzCD*::Tn*5lac*Ω536 Δ*pilT*	This work
SA3029	*frzCD*::Tn*5lac*Ω536 Δ*pilT/*P_pilA_*–yfp–pilT* (pIB75)	This work
SA3043	Δ*pilB,*Δ*pilT/*P_pilA_*–yfp–pilT* (pIB75)	This work
SA3045	Δ*pilT/*P_pilA_*–yfp–pilT* (pIB75)	This work
SA3046	Δ*pilM/*P_pilA_*–yfp–pilM* (pCS8)	This work
SA3049	Wild type*/*P_pilA_*–pilT* (pSL104)	This work
SA3057	*frzCD*::Tn*5lac*Ω536 Δ*pilM*	This work
SA3059	*frzCD*::Tn*5lac*Ω536 Δ*pilM/*P_pilA_*–yfp–pilM* (pCS8)	This work
SA3064	Wild type*/*P_pilA_*–yfp–pilT* (pIB75)	This work

a All plasmids were integrated at the Mx8 *attB* site. In P_pilA_ constructs the *pilM* and *pilT* alleles were transcribed from the *pilA* promoter.

### Live-cell imaging and data analysis

For phase-contrast and fluorescence microscopy, exponential cultures of *M. xanthus* were grown to a density of 7 × 10^8^ cells per ml in liquid CTT medium at 32°C, transferred to a microscope slide and immediately observed in a Leica DM6000B microscope using a Leica Plan Apo 100×/NA 1.40 phase-contrast oil objective and visualized with a Roper Photometrics® Cascade II 1024 camera. For fluorescence microscopy, a Leica YFP filter (excitation range 490–510 nm, emission range 520–550 nm) was used. Images were recorded and processed with Image-Pro® 6.2 (MediaCybernetics). Processed images were arranged in Adobe Photoshop 6 (Adobe Systems). For time-lapse recordings, cells were spotted on a thin 0.7% agar-pad buffered with A50 starvation buffer (10 mM MOPS, pH 7.2, 10 mM CaCl_2_, 10 mM MgCl_2_, 50 mM NaCl) on a glass slide and immediately covered with a coverslip. After 30 min at room temperature, cells were imaged at 30 s intervals. Quantification of fluorescence was performed as follows. The average fluorescence intensities (arbitrary units) of polar clusters (Fig. 1C) and of the cytoplasmic region between the polar clusters (Fig. 1C) were measured using the region measurement tool in Metamorph 7.0r.2 (Molecular Devices). The average fluorescence intensity of the cytoplasmic region was subtracted from the average fluorescence intensities of the polar clusters. The background-subtracted values were corrected for area size generating integrated intensities, which were used to calculate ratios between polar signals or plotted as a function of time. For each strain, at least 100 cells were analysed.

### Immunofluorescence microscopy and data analysis

Immunofluorescence microscopy was performed essentially as described (Mignot *et al.*, 2005). Briefly, *M. xanthus* cells were either fixed directly from suspension or harvested, re-suspended in 1% CTT medium to a calculated density of 7.0 × 10^9^ cells per ml, and placed (10 μl aliquots of cells) on 1.0% agar supplemented with 0.5% CTT in order to analyse cells on a surface. After 3–4 h incubation at 32°C, cells were harvested from the plate by washing with 1% CTT medium mixed with fixing solution. Cells were fixed with 3.2% (PilC and PilM) or 1.6% paraformaldehyde (PilQ, PilB and PilT) and 0.008% glutaraldehyde for 20 min on freshly prepared poly l-Lysine-treated 12-well diagnostic slides (Thermo Scientific). Cells were permeabilized with GTE buffer (50 mM glucose, 20 mM Tris, 10 mM EDTA, pH 7.5) for 4 min and probed with relevant affinity-purified polyclonal antibodies at 4°C overnight in PBS buffer (137 mM NaCl, 2.7 mM KCl, 10 mM Na_2_HPO_4_, 1.8 mM KH_2_PO_4_, pH 7.4) supplemented with 2% BSA. DyLight 547-conjugated goat anti-rabbit antibodies (Perbio Science) were used as a secondary antibody. Slow Fade Anti Fade Reagent (Molecular Probes) was added to each well. Cells were observed in a Leica DM6000B microscope using a Leica Plan Apo 100×/NA 1.40 phase-contrast oil objective and visualized with a Leica DFC 350FX camera. For fluorescence microscopy, a Leica Y3 filter (excitation range 530–560 nm, emission range 570–650 nm) and a Leica GFP filter (excitation range 450–490 nm, emission range 500–550 nm) were used. Processed images were arranged in Adobe Photoshop 6. Quantification of fluorescence signals was performed as described for YFP–PilM/T. For each strain, at least 100 cells were analysed.

### FRAP experiments and data analysis

For FRAP experiments *M. xanthus* cells were grown as described, transferred to a microscope slide, covered with a polystyrene-covered cover glass and sealed. After 30–60 min at room temperature, cells were observed in a Nikon Eclipse TE 2000-E microscope (Nikon) with a Nikon CFI Plan Fluor 100×/NA 1.30 oil immersion objective. For photobleaching, a 514 nm laser beam of an argon ion laser (Melles Griot Laser Group) was focused in the image plane of the microscope. The cytoplasmic region (Fig. 1C) was pre-bleached for 3 s in order to decrease non-polar fluorescence signals. Subsequently, an entire polar region was bleached for 1 s, and recovery of the fluorescence signal followed for 120 s, with time intervals of 3 s between frames. Images were recorded with a Roper Photometrics® Cascade II 512 camera. Images were processed with NIS Elements AR 2.30 (Nikon). Data analysis was performed as follows. Integrated fluorescence intensities (arbitrary units) of polar YFP–PilT clusters and of the cytoplasmic region (Fig. 1C) were measured using the region measurement tool in Metamorph 7.0r.2. Integrated intensities were plotted as a function of time. Because the total fluorescence signal over individual cells did not change over time after bleaching of a polar region (Fig. 7B and C), the data were not corrected for continuous bleaching. A total of 10 cells were analysed.
