# Supplementary material for: Regulation of the type IV pili molecular machine by dynamic localization of two motor proteins
Source: Mol Microbiol. 2009 Oct 6;74(3):691–706. doi: 10.1111/j.1365-2958.2009.06891.x (PMC2784877; doi:10.1111/j.1365-2958.2009.06891.x)
Supplement: Supplementary file 1 [file mmi0074-0691-SD1.pdf]

Supplementary Materials for

Regulation of the type IV pili molecular machine by dynamic localization of two motor proteins

Iryna Bulyha, Carmen Schmidt, Peter Lenz, Vladimir Jakovljevic, Andrea Höne, Berenike Maier, Michael Hoppert, and Lotte Søgaard-Andersen

This PDF file includes:

Theoretical model for dynamic PilT localization.

Legends to Supplementary Figures.

Supplementary Experimental Procedures.

References.

Table S1.

Figure S1-S4.

Theoretical model for dynamic PilT localization

As shown in the main text, diffusion from pole to pole is expected to take ~7s for a PilT monomer and ~12s for a PilT hexamer. On the other hand, as can be seen from Fig. 7 it takes 50±10s until the intensity of the bleached pole reaches the new plateau value. This suggests that the recovery is not limited by diffusion of PilT in the cytoplasm. Based on this assumption we have developed a theoretical model for the dynamics of PilT in the FRAP experiments. In our model, PilT can bind to either the leading or the lagging pole. Then, the time-dependence of the concentration of bound protein at the lagging pole (c_l), the concentration of bound protein at the leading pole (c_r) and the concentration of protein in the cytoplasm (c_f) obey the following rate equations:

$$\frac{dc_l}{dt} = l_+ c_f - l_- c_l, \quad (1)$$

$$\frac{dc_r}{dt} = r_+ c_f - r_- c_r, \quad (2)$$

$$\frac{dc_f}{dt} = -(l_+ + r_+) c_f + l_- c_l + r_- c_r. \quad (3)$$

Here, $l_-(l_+)$ are the off (on) rates at the lagging pole and $r_-(r_+)$ the off (on) rates at the leading pole. As we show below this simple model is able to explain all the experimental FRAP data. We also tried a simpler model for which $l_- = r_-$ and $l_+ = r_+$. However, this model is not able to explain the 'crossing' of fluorescence intensity as observed after bleaching of the (larger) lagging pole (Fig. 7B) (see below).

The steady state value of c_f is given by

$$c_f = \frac{l_- c_l + r_- c_r}{l_+ + r_+}. \quad (4)$$

This implies

$$c_r(t) + c_l(t) = c_b = const. \quad (5)$$

i.e. the total amount of bound protein (bound either at the leading or lagging pole) is constant in time as was observed experimentally (Fig. 7BC).

In a typical FRAP experiment one of the poles, say the lagging pole, is bleached giving rise to a concentration $c_l(0)$ of fluorescent bound protein. As explained in the main text the fluorescence signal of the bleached pole recovers giving rise to a new steady state (reached as time t approaches ∞) where the concentrations of fluorescent bound protein at lagging (leading) pole is denoted by $c_l(\infty)(c_r(\infty))$.

By integrating Eqs. (1)-(3) the dynamics of this recovery process can be obtained (by assuming for simplicity that only a small fraction of the cytoplasmic protein is bleached and that the amount of bleached protein leaving the bleached pole is much smaller than the amount of protein in the cytoplasm)

$$c_l(t) = e^{-\alpha t}[c_b(e^{\alpha t} - 1)\frac{1}{1+\beta} + c_l(0)]. \tag{6}$$

$$c_r(t) = e^{-\alpha t}[c_b\frac{e^{\alpha t}}{1+1/\beta} + \frac{c_b}{1+\beta} - c_l(0)]. \tag{7}$$

Here,

$$\alpha = \frac{l_+ r_- + l_- r_+}{l_+ + r_+} \tag{8}$$

$$\beta = \frac{l_- r_+}{l_+ r_-}. \tag{9}$$

It is important to note that the dynamics by which the PilT concentrations at both the bleached and the non-bleached pole reach their new steady state values is characterized by the same rate constant α .

The constant β can be read off from the experimental new steady state fluorescent protein concentrations at the two poles

$$c_l(\infty) = \frac{c_b}{1+\beta} \tag{10}$$

$$c_r(\infty) = \frac{c_b}{1+1/\beta}. \tag{11}$$

Similarly, α can be determined from the experimental data by plotting the time-dependent approach to the new steady state values

$$\frac{c_l(t)-c_l(\infty)}{c_l(\infty)}=e^{-\alpha t}(\frac{c_l(0)}{c_b}(1+\beta)-1), \tag{12}$$

$$\frac{c_r(t)-c_r(\infty)}{c_r(\infty)}=e^{-\alpha t}(1-\frac{c_l(0)}{c_b}(1+1/\beta)). \tag{13}$$

We used the mathematical model to determine the values of α and β from the FRAP data. α is determined from Eqs. (12) and (13) and β can be determined from Eqs. (10) and (11). By analyzing FRAP data as those shown in Fig. 7 for 10 cells, we find that $\alpha=0.02\pm0.01 s^{-1}$ and $\beta=0.64\pm0.46$. For both values the cell to cell variations are comparable to the errors in determining the values for a single cell. The half-life time of the clusters (given by $\ln(2)/\alpha$) is calculated to 40±20s.
The mean waiting time for binding of PilT is given by

$$\frac{1}{2}(\frac{1}{l_+}+\frac{1}{r_+})=\frac{1}{2}\frac{r_-}{r_+}\frac{1+\beta}{\alpha} \tag{14}$$

where Eq. (6) has been used. For the ratio between on and off rates one has

$$\frac{r_-}{r_+}=\frac{c_f}{c_r}=\frac{c_f}{c_b}(1+1/\beta), \tag{15}$$

where Eqs. (2) and (9) have been used. The ratio between the concentrations of free and bound PilT is found to be $c_b/(c_b+c_f)=0.7\pm0.2$ (where the polar regions occupy 20% of the cellular volume). This is in good agreement with the immuno-fluorescence data which yields $c_b/(c_b+c_f)=0.58\pm0.08$. With these immuno-fluorescence data the mean waiting time for binding of PilT becomes of the order of 80s. Dissociation of PilT from the poles is even slower than association. Since the concentration of PilT is larger at the poles than in the cytoplasm one has $r_-<r_+$ and $l_-<l_+$ and therefore

$$\frac{1}{2}(\frac{1}{l_-}+\frac{1}{r_-})\geq\frac{1}{2}(\frac{1}{l_+}+\frac{1}{r_+}). \tag{16}$$

Legends to Supplementary Figures

Fig. S1. Immunoblot of PilQ accumulation.

Cells from the exponentially growing cultures were harvested, total protein separated by SDS–PAGE (protein from 5 $\times 10^7$ cells loaded per lane), and analyzed by immunoblotting. Strains used (left to right): DK1622, DK8615. The blot was probed with rabbit anti-PilQ antibodies. The three proteins recognized by the anti-PilQ antibodies have sizes of approx. 250 kDa, 98 kDa and 50 kDa. These proteins correspond to heat and detergent resistant PilQ multimers (black arrow), PilQ monomers (dark grey arrow) and a degradation product of PilQ (light grey arrow), respectively (Nudleman et al., 2006). Migration of molecular size markers is indicated on the left.

Figure S2. Immunoblot of PilC accumulation.

Cells from exponentially growing cultures were harvested and samples analyzed as in Fig. S1. Strains used (left to right): DK1622, DK10417. The blot was probed with rabbit anti-PilC antibodies. PilC is indicated by the arrow. Migration of molecular size markers is indicated on the left.

Figure S3. Analysis of PilC and PilM

(A) Subcellular localization of PilC and PilM. Total cell extract of vegetative cells (T) was separated into fractions enriched for soluble (S), inner membrane (IM), and outer membrane (OM) proteins. The first lanes contain total cell extract from wild-type cells (DK1622) and the second lanes contain total cell extract from (top panel to bottom panel) SA3002 (Δ*pilM*), DK8615 (Δ*pilQ*), DK10416 (Δ*pilB*) and DK10417 (Δ*pilC*) cells. Protein from 10^8 cells was added per lane and analyzed using antibodies (top panel to bottom panel) against PilM, PilQ, PilB or PilC.

(B) Immunoblot of PilM and YFP-PilM accumulation. Cells from exponentially growing cultures were harvested and samples analyzed as in Fig. S1. Strains used (left to right): DK1622, SA3002, and SA3046. Blot on the left was probed with rabbit anti-PilM antibodies, and blot on the right with monoclonal anti-GFP antibodies, which also recognize YFP. PilM and YFP-PilM proteins are indicated with the arrows. Migration of molecular size markers is indicated on the left.

(C) YFP-PilM complements the motility defect in a Δ*pilM* mutant. Cells were incubated at 32˚ for 24h on 0.5% agar supplemented with 0.5% CTT, and visualized with a Leica MZ8

stereomicroscope. Scale bar: 5 mm. We analyzed the strains for T4P-dependent motility on 0.5% agar, which favors T4P-dependent motility. The wild type DK1622 formed colonies with large rafts of cells at the edge typical of T4P-dependent motility whereas SA3002 (Δ*pilM*) did not form rafts at the edge. However, SA3046 (Δ*pilM/pilM-yfp*) displayed a motility phenotype similar to that of the wild type.

<u>Figure S4. Analysis of YFP-PilT.</u>

(A) YFP-PilT complements motility phenotypes in Δ*pilT* mutant. Cells were incubated at 32˚ for 24h on 0.5% agar supplemented with 0.5% CTT, and visualized with a Leica MZ8 stereomicroscope. Scale bar: 5 mm. See Fig. S3C for a description of the assay.

(B) Immunoblot of PilT and YFP-PilT accumulation. Cells from exponentially growing cultures were harvested and samples analyzed as in Fig. S1. Strains used (left to right): DK1622, DK10409, SA3049, SA3045, SA3064. Blot on the left was probed with rabbit anti-PilT antibodies, and blot on the right with monoclonal anti-GFP antibodies. PilT and YFP-PilT proteins are indicated. Grey arrow indicates breakdown product of YFP-PilT of approx. 50 kDa. Migration of molecular size markers is indicated on the left. YFP-PilT, YFP and PilT have calculated molecular masses of 68, 26 and 42 kDa, respectively.

(C) YFP-PilT localization in moving cells. Cells of SA3045 were grown exponentially in CTT, transferred to a thin 0.7% agar pad on a microscope slide, and imaged by fluorescence microscopy at 60s intervals. The SA3045 cell reversed a total of four times within 15 min, i.e. from 4:00 to 5:00 min, 8:00 to 9:00 min, 10:00 to 11:00 min and from 14:00 to 15:00 min. White arrows indicate the direction of movement.

(D) Quantitative analysis of polar YFP-PilT fluorescence signals. Integrated fluorescence intensities (arbitrary units) of the two background subtracted polar clusters in the cells in (C) plotted as a function of time.

Supplementary Experimental Procedures

Construction of plasmids and strains. Plasmids were propagated in *E. coli* TOP10 (F^-, *mcrA*, Δ(*mrr-hsd*RMS-*mcr*BC), φ80*lac*ZΔM15, Δ*lac*X74, *deo*R, *rec*A1, *ara*D139, Δ(*ara-leu*)7679, *gal*U, *gal*K, *rps*L, *end*A1, *nup*G) unless otherwise stated. Primers used are listed in Table S1 in Supplemental data. All DNA fragments generated by PCR were verified by sequencing. DK1622 was used as the wild type throughout. All strains constructed were confirmed by PCR. The in-frame deletion of *pilM* (SA3002) was generated as described (Shi et al., 2008) using the primers oPilM-ABCD (A list of all primers used in this work is included in Table S1) after cloning of the appropriate constructs in pBJ114.

To construct pIB75, which contains P*pilA-yfp-pilT,* the *pilT* gene was amplified using the primers opilT-4 and opilT-7 and pSL107 as a template giving rise to the full-length *pilT* with an additional 10 aa linker at the 5'-end (S. Leonardy, pers. communication). The PCR product was digested with *EcoRI* and *HindIII*, and cloned in in pBluescript II SK- generating pIB71. The *yfp* gene was amplified by PCR using the primers oYFP-1 and oYFP-2 and pSW105-YFP as a template (V. Jakovljevic, pers. communication). The PCR product was digested with *SpeI* and *EcoRI*, and cloned in-frame with *pilT* in pIB71 generating pIB72. The *SpeI*-*HindIII* fragment from pIB72 was then re-cloned into pSW105, which contains the *pilA* promoter giving rise to pIB73. Finally, the *NdeI*-*HindIII* fragment of pIB73 containing P*pilA-yfp-pilT* was cloned into pSWU30, generating pIB75.

To construct pIB74, which contains P*pilA-yfp-pilT*^{E205A}, plasmid DNA of pSL4TWalkerB (Jakovljevic et al., 2008) was digested with *XbaI* and *HindIII*. The resulting fragment, which contained *pilT* with the E205A substitution, was cloned into pIB75 instead of the wild-type *pilT* gene, generating pIB74.

To construct pCS8, which contains a P*pilA-yfp-pilM* construct, *M. xanthus* chromosomal DNA was amplified with the primers opilM-3 and opilM-4 giving rise to a *pilM* gene extending from position +1 to +1188. The PCR product was digested with *XbaI* and *HindIII* and cloned into pIB75 instead of *pilT* gene generating pSC8.

Plasmids containing *yfp-pilT* and *yfp-pilM* alleles were integrated by site-specific recombination at the Mx8 attB site on the chromosome. Strains containing plasmids integrated at the *attB* site were constructed by electroporation of the plasmid into the relevant strain (Kashefi and Hartzell, 1995) followed by selection to the relevant antibiotic. All strains were verified by PCR.

To construct pPilC-CD1, which encodes a His6-tagged truncated PilC protein extending from residue 1-185, a *pilC* PCR fragment generated with the primers oPilC1 and oPilC5 (covering the N-terminal bases 1 to 556 of *pilC*) was digested with *BamHI* and *EcoRI* and cloned into pET-24b+ vector (Novagen).

To construct pIB49, which encodes His6-tagged full-length PilQ, a *pilQ* fragment was generated with the primers oPilQ-7 and oPilQ-8, digested with *PstI* and *HindIII* and cloned into pET-45b(+) (Novagen).

To construct pCS3, which encodes His6-tagged full-length PilM, a full-length *pilM* PCR product was generated using the primers oPilM-1(with an *EcoRI* site followed by six histidine codons and the first 18 nucleotides of *pilM*) and oPilM-2 (containing the last 21 nucleotides of *pilM* and a *HindIII* site) and cloned into pBluescript II SK- giving rise to pIB35. The *EcoRI-HindIII* fragment was recloned into the pUHE24-2 expression vector (Lanzer and Bujard, 1988) to give pCS3.

Antibody generation and immunoblots. pPilC-CD1 and pIB49 were propagated in *E. coli* Rosetta 2 [F-*ompT hsdS*$_B$ *(r*$_B^-$ *m*$_B^-$*) gal dcm p*RARE2] (Novagen). pCS3 was propagated in *E. coli* JM109 [F' *traD36 proA*$^+$*B*$^+$ *lacI*^{q} Δ*lac*ZM15/Δ(*lac-proAB*) *glnV44 e14- gyrA96 recA1 relA1 endA1 thi hsdR17*] (New England Biolabs) containing the plasmid pMS421, which contains the *lacI*^{q} allele (Grana et al., 1988). PilC$^{1-185}$-His$_6$ was purified under native conditions as described (Rasmussen et al., 2005). His$_6$-PilQ and His$_6$-PilM were purified under denaturing conditions as described (Jakovljevic et al., 2008). Purified proteins were used to immunize a rabbit using standard procedures (Sambrook et al., 1989). Western blotting was performed using standard procedures (Sambrook et al., 1989) with polyclonal rabbit anti-PilQ, anti-PilC, anti-PilM, anti-PilB (Jakovljevic et al., 2008) and anti-PilT (Jakovljevic et al., 2008) antibodies and peroxidase-conjugated goat anti-rabbit immunoglobulin G secondary antibodies as recommended by manufacturer (Roche). Anti-PilQ (anti-PilT) antibodies were affinity-purified using His$_6$-PilQ (His$_6$-PilT) bound to a nitrocellulose membrane. Anti-PilM antibodies were purified with cell lysate from Δ*pilM* strain. For detection of YFP-tagged proteins, monoclonal anti-GFP mouse antibody (Roche) and peroxidase-conjugated rabbit anti-mouse immunoglobulin G secondary antibody (DakoCytomation) were used. Blots were developed using the Supersignal West Pico chemiluminescence reagent (Pierce).

Cell fractionation. Biochemical fractionation of cells was done as described (Lobedanz and Søgaard-Andersen, 2003). Briefly, cells were grown in CTT. To separate inner membrane and outer membrane proteins from soluble proteins (Lobedanz and Søgaard-Andersen, 2003), cells were resuspended in 50 mM Tris-HCl pH 7.6 supplemented with Complete Mini Protease Inhibitor Cocktail (Roche) (protease inhibitors) as recommended by the supplier (equivalent to 1x concentration of protease inhibitors) and lysed by sonication. Cell debris was removed by centrifugation. The supernatants were centrifuged at 45,000 *g* for 1 hr at 4°C. The resulting supernatants are enriched in soluble proteins. Pellets containing the crude envelope fractions were resuspended in 50 mM Tris-HCl pH 7.6, 2% Triton X-100 supplemented with protease inhibitors, and subjected to ultracentrifugation as described. The resulting supernatant is enriched in inner membrane proteins, and the pellet is enriched in outer membrane proteins. All fractions were analyzed by immunoblotting. As controls for proper fractionation, fractions were tested with antibodies against PilB in the cytoplasm (Jakovljevic et al., 2008) and PilQ in the outer membrane (Nudleman et al., 2006).

Transmission electron microscopy (TEM). TEM was used to visualize T4P as described (Jakovljevic et al., 2008). Significance was determined using the *t* test.

References

Grana, D., Gardella, T., and Susskind, M.M. (1988). The effects of mutations in the ant promoter of phage P22 depend on context. Genetics *120*, 319-327.

Jakovljevic, V., Leonardy, S., Hoppert, M., and Søgaard-Andersen, L. (2008). PilB and PilT are ATPases acting antagonistically in type IV pili function in *Myxococcus xanthus*. J Bacteriol *190*, 2411-2421.

Kashefi, K., and Hartzell, P.L. (1995). Genetic suppression and phenotypic masking of a *Myxococcus xanthus frzF* defect. Mol Microbiol *15*, 483-494.

Lanzer, M., and Bujard, H. (1988). Promoters largely determine the efficiency of repressor action. P Natl Acad Sci USA *85*, 8973-8977.

Lobedanz, S., and Søgaard-Andersen, L. (2003). Identification of the C-signal, a contact dependent morphogen coordinating multiple developmental responses in *Myxococcus xanthus*. Genes Dev *17*, 2151-2161.

Nudleman, E., Wall, D., and Kaiser, D. (2006). Polar assembly of the type IV pilus secretin in *Myxococcus xanthus*. Mol Microbiol *60*, 16-29.

Rasmussen, A.A., Porter, S.L., Armitage, J.P., and Søgaard-Andersen, L. (2005). Coupling of multicellular morphogenesis and cellular differentiation by an unusual hybrid histidine protein kinase during fruiting body morphogenesis in *Myxococcus xanthus*. Mol Microbiol *56*, 1358-1372.

Sambrook, J., Fritsch, E.F., and Maniatis, T. (1989). Molecular Cloning. A Laboratory Manual (Cold Spring Harbor, N.Y., Cold Spring Harbor Laboratory Press).

Shi, X., Wegener-Feldbrügge, S., Huntley, S., Hamann, N., Hedderich, R., and Søgaard-Andersen, L. (2008). Bioinformatics and experimental analysis of proteins of two-component systems in *Myxococcus xanthus*. J Bacteriol *190*, 613-624.

Table S1. Primers used in this work

Name	Nucleotide sequence (5'-3')[1]
oPilM-A	atcgg**aagctt**GGGCTCACCGCAGAGGCC
oPilM-B	GCCCGGGCGgtgcctgga
oPilM-C	TCCAGGCACcgccgggc
oPilM-D	atcgg**gaattc**TGCTGTCTTGCTGTCCG
PilC1	atcgtc**ggatcc**AGCAGCCCCAGCAGTGAAGTC
PilC5	cgtagt**aagctt**CTACATCGCGCTCTTGACCTTC
opilT-4	atcgg**aagctt**CTAACGACCACCCGCTCC
opilT-7	atc**gaattc**GGATCGGCCGGCTCCGCCGCCGGCTCCGGCTCTGGA
oYFP-1	atc**actagt**TGGTGAGCAAGGGCGAG
oYFP-2	atc**gaattc**CTTGTACAGCTCGTCCAT
opilM-3	atcgg**aagctt**TCAGGCCAGCTTGTCGCC
opilM-4	atcgg**tctaga**GTGGTGCGAGGCTCCCGT
oPilQ-7	atcgg**ctgcag**ATGCCGACCTTTACCGTG
oPilQ-8	atcgg**aagctt**TTACAGAGTCTGCGCAAT
oPilM-1	atcgg**gaattc**attaagaggagaaattaactatg**catcaccatcaccatcac**GTGCGA GGCTCCCGTCCG
oPilM-2	atcgg**aagctt**TCAGGCCAGCTTGTCGCCCGG

[1] Sequences in bold indicate restriction sites used for cloning. Sequences in upper case indicate sequences in the respective genes. Sequences in lower case indicate additional sequences required for cloning

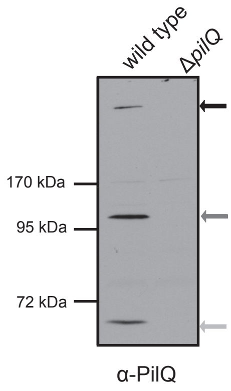

Figure S1: Bulyha et al.

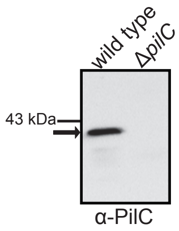

Figure S2: Bulyha et al.

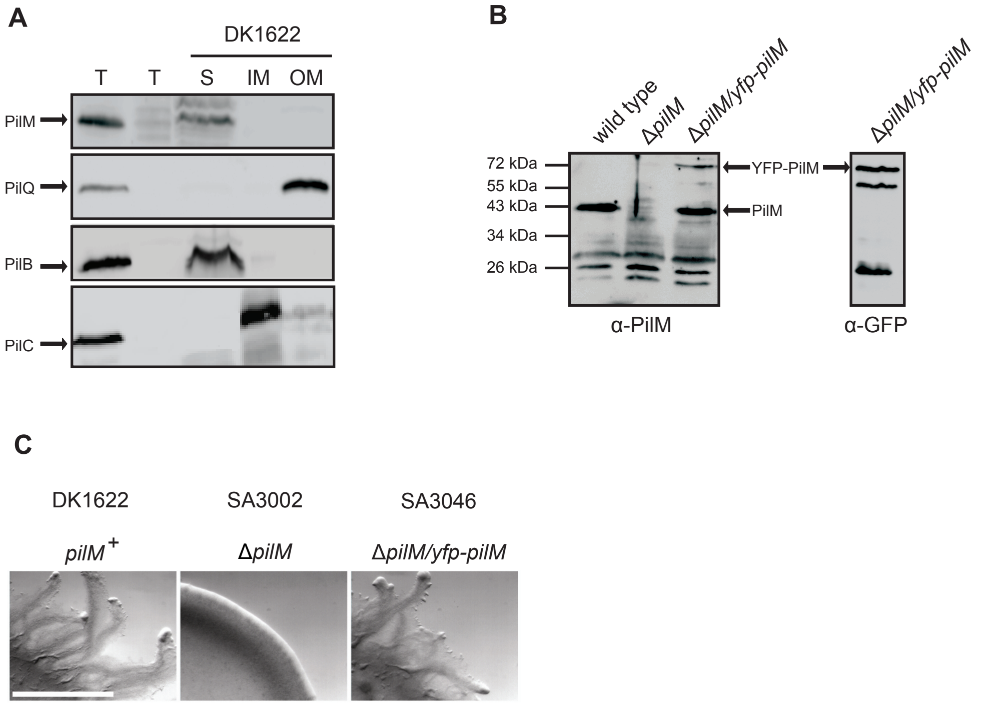

Figure S3: Bulyha et al

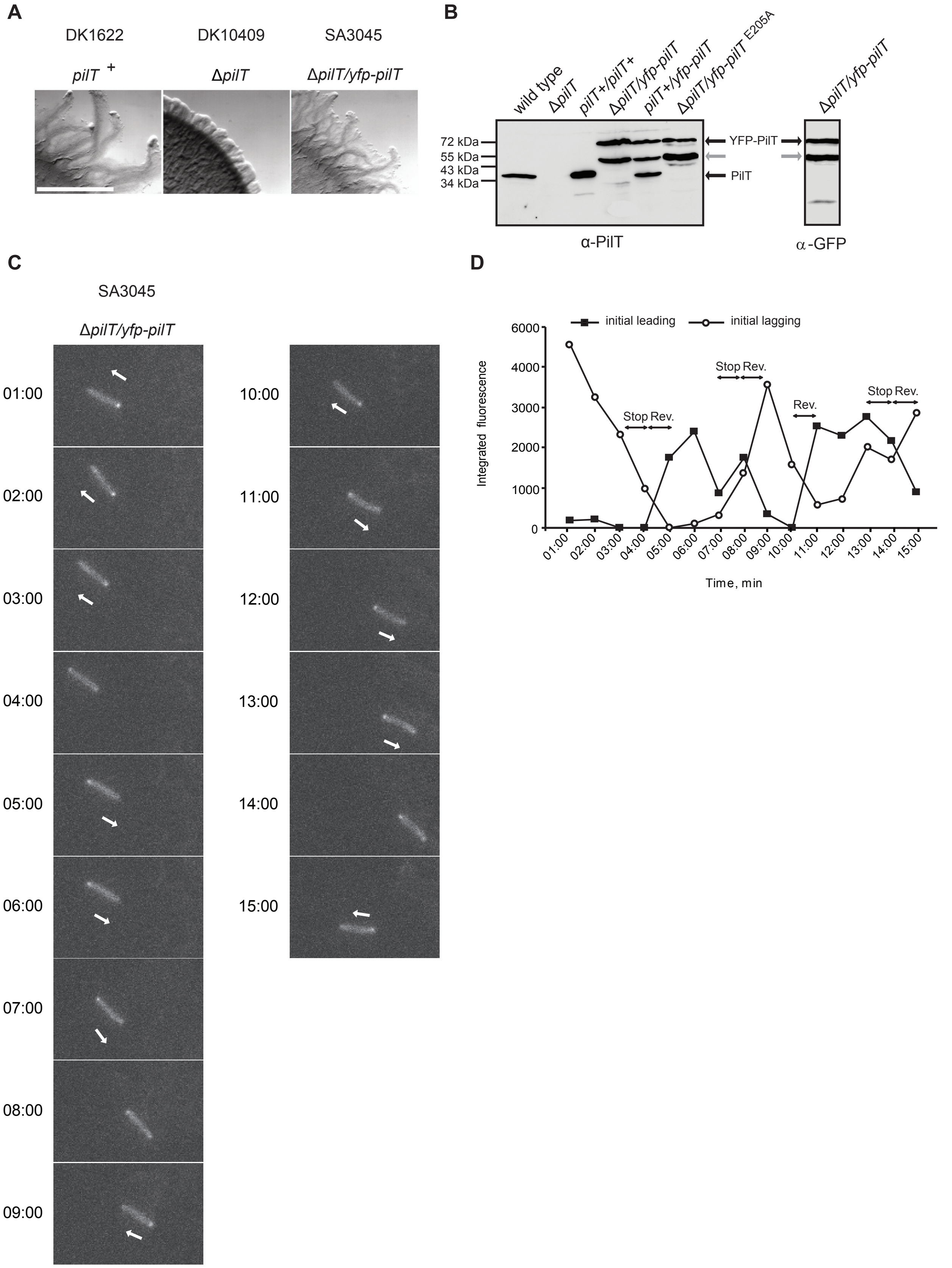

Figure S4: Bulyha et al.
